# Breaking Seed Dormancy during Dry Storage: A Useful Tool or Major Problem for Successful Restoration via Direct Seeding?

**DOI:** 10.3390/plants9050636

**Published:** 2020-05-16

**Authors:** Carol C. Baskin, Jerry M. Baskin

**Affiliations:** 1 Department of Biology, University of Kentucky, Lexington, KY 40506-0225, USA; 2 Department of Plant and Soil Science, University of Kentucky, Lexington, KY 40546-0321, USA; jerry.baskin@yahoo.com

**Keywords:** afterripening, nondormant seeds, physiological dormancy, secondary dormancy, seed moisture content

## Abstract

To facilitate the restoration of disturbed vegetation, seeds of wild species are collected and held in dry storage, but often there is a shortage of seeds for this purpose. Thus, much research effort is expended to maximize the use of the available seeds and to ensure that they are nondormant when sown. Sowing nondormant (versus dormant) seeds in the field should increase the success of the restoration. Of the various treatments available to break seed dormancy, afterripening, that is, dormancy break during dry storage, is the most cost-effective. Seeds that can undergo afterripening have nondeep physiological dormancy, and this includes members of common families such as Asteraceae and Poaceae. In this review, we consider differences between species in terms of seed moisture content, temperature and time required for afterripening and discuss the conditions in which afterripening is rapid but could lead to seed aging and death if storage is too long. Attention is given to the induction of secondary dormancy in seeds that have become nondormant via afterripening and to the biochemical and molecular changes occurring in seeds during dry storage. Some recommendations are made for managing afterripening so that seeds are nondormant at the time for sowing. The most important recommendation probably is that germination responses of the seeds need to be monitored for germinability/viability during the storage period.

## 1. Introduction

Seeds of many wild species are collected and placed in dry storage because they are needed for habitat restoration projects [1]. At some point in time, the stored seeds will be sown in the field in an attempt to establish new populations [2] or in nurseries to generate plants for out-planting to sites that need to be restored [3,4,5]. The demand for seeds for restoration is enormous, and it has become very clear that the quantities of seeds needed for these endeavors cannot be obtained solely by collecting them from wild plants [2,6,7,8,9,10,11]. Various solutions to the seed-shortage problem have been proposed, including the development of seed production areas for wild species [9,12,13,14]; establishment of community seed banks [6]; and formation of regional [15] and national [16] strategies to deal with the collection, production and management of seeds needed for restoration. Further, plans need to be developed to maximize the use of seeds that have been collected [17], for example, to ensure that seeds are viable and nondormant when sown, and León-Lobos et al. [11] advocate that additional research on seed dormancy-breaking and germination requirements needs to be conducted.

Although restoration can be attempted/achieved by direct sowing of seeds in the field and by transplanting juvenile plants to the site, the most cost-effective method of restoration is direct seeding [18,19,20,21]. Direct seeding in the field avoids the expenses for pots, soil, water, transportation to the field site and wages for helpers to care for young plants and to transplant juveniles from pots into soil at the restoration site. However, sowing seeds in the field may not result in a high percentage of emerged seedlings [22]. For example, seedlings emerged from only 8%–10% of the caryopses of the grasses *Triodia epactia* S.W.L. Jacobs and *T. wiseana* C.A. Gardner sown in the field in the Pilbara region of NW Western Australia, and this was in the water-addition (4 × 24 mm of water) treatment; no seedlings emerged in the non-watered control [23]. Seedling emergence from caryopses of the grasses *Agropyron desertorum* (Fisch. ex Link) Schult., *Elymus elymoides* (Raf.) Swezey and *Pseudoroegenaria spicata* (Pursh) A. Löve sown in the field in the Great Basin in Eastern Oregon (USA) was only 17% and 7% in tilled plots and burned sites, respectively [24]. However, direct seeding was effective in a neotropical savanna when the area was plowed and heavily seeded [20]. 

There are many reasons why seeds sown in the field may not germinate, including lack of sufficient soil moisture at the time temperatures are favorable for germination [25], light/dark requirements not fulfilled (for example [26]) and destruction of seeds by animals [27]. Thus, seeds usually are sown in the field when environmental factors are perceived to be favorable for germination, and sometimes experimental seed-sowing plots are watered at least once, for example [28]. Another important reason for a lack of germination when seeds are sown in the field is that they are dormant. It has been suggested that mixtures of species, some with nondormant seeds and others with dormant seeds, sown at the same time at a restoration site might result in the vegetation being dominated by plants of the species with nondormant seeds [29].

Although freshly-matured seeds of some species are nondormant and require no dormancy-breaking treatments [30], seeds of many species are dormant at maturity. Thus, much research effort has been devoted to finding effective treatments to break dormancy prior to sowing. Pretreatments to break the dormancy of seeds with water-impermeable seed coats (physical dormancy, PY), include mechanical scarification [31], chemical scarification (usually with concentrated sulfuric acid) [32] and dipping in hot or boiling water [33,34]. If large quantities of seeds with PY need to be made water-permeable, this can be done by various machines that have been developed to scarify seeds (see [35,36,37]). Seeds with water-permeable seed coats may be given cold (moist) stratification [30]; treated with karrikinolide (KAR_1_), gibberellic acid-3 (GA_3_) [38,39] or sodium hypochlorite [40]; scarified [40]; or given a heat treatment [41]. Also, removing the covering structures of grass caryopses can promote germination [42,43,44]. 

Afterripening, or dormancy-break during dry storage, is another way in which dormancy of seeds to be used for restoration can be broken [29]. Originally, “afterripening” was used to describe dormancy-breaking that occurred after seed dispersal [45]. Thus, the term has been used to describe the growth of underdeveloped embryos that must occur in seeds of some species before they can germinate [46] and dormancy-break during incubation at cold (moist) conditions [47]. Since at least the 1950s, “afterripening” has been used primarily to describe dormancy-break during dry storage of seeds [48,49], and this is how we use it in this review.

Many plant researchers are aware that a period of dry storage at room temperatures may break seed dormancy (for example [41,50,51,52,53,54]), but the potential of this method to break seed dormancy of species to be used in restoration projects has not been fully realized. Clearly, if seeds are water-permeable and will afterripen during dry storage, this is a cost-effective method to break dormancy prior to sowing. However, depending on the species, afterripening requirements can vary, and an extended afterripening treatment could lead to induction into secondary dormancy or to seed aging (loss of vigor) and death (see below). The purpose of this paper is to provide an overview of dormancy break during dry storage and make recommendations with regard to the management of afterripening so that it is a useful tool and not a major headache for restoration.

## 2. Seeds That Can Afterripen 

In all major vegetation zones on earth, there are more species with dormant than nondormant seeds, except in evergreen tropical rainforests, where the percentages of species with dormant and nondormant seeds are 51.4% and 48.6%, respectively [55]. There are five classes of dormancy: morphological, morphophysiological, physiological, physical and combinational (physical + physiological). In all vegetation zones on earth, physiological dormancy (PD) is the most common class. Seeds with PD have fully developed embryos that have a physiological inhibiting mechanism for germination. There are three levels of PD (nondeep, intermediate and deep), and nondeep is by far the most common [55]. Nondeep PD is the kind of dormancy in seeds of various families such as Amaranthaceae, Asteraceae, Brassicaceae, Caryophyllaceae, Euphorbiaceae, Lamiaceae, Myrtaceae, Poaceae, Plantaginaceae, Proteaceae, Scrophulariaceae and Solanaceae. An important characteristic of seeds with nondeep PD is that dormancy-break can occur during dry storage; that is, they can afterripen. Starting with freshly-matured seeds, afterripening is detected by testing samples of seeds for germination over a range of conditions after various periods of storage.

During afterripening, germination percentages and rates (speed) and the range of conditions over which seeds will germinate may increase [56,57,58]. For example, during afterripening seeds of many winter annuals exhibit an increase not only in percentages and rates of germination but also in the maximum temperature at which they can germinate [59]. In terms of parameters of the hydrothermal time model, the base temperature (T_b_) can shift, for example in the summer annual *Polygonum aviculare* L. as dormancy break occurs [60]. Also, during afterripening the response of seeds to light/dark and water stress can change. For example, seeds of *Bromus diandrus* Roth stored dry at room temperature for two months germinated to 80–100% in the dark at 10, 15, 20 and 25 °C at water potentials of 0, −0.2, and −0.4 MPa; however, those in the light germinated to only 0–10%, regardless of test conditions [61]. Following 12 months of afterripening, seeds of this species incubated in the dark at 10, 15, 20 and 25 °C at 0, −0.2, −0.4 and −0.8 MPa germinated to 95–100%, while those incubated in light at 10, 15 and 20 °C at 0 and −0.2 MPa germinated to 85–95%. Germination in the light at 10, 15 and 20 °C at −0.4 MPa was about 50%, 50% and 90%, respectively. Thus, afterripening increased the ability of seeds to germinate in the light and at increased water stress. That is, the hydrotime parameters of seeds were modified by afterripening, resulting in a shift of the base water potential (Ψ_b_) for germination to more negative values [61]. The base water potential for germination also became more negative for seeds of *Bromus tectorum* L. as they afterripened [62]. 

Afterripening has been reported in seeds that remain on dead mother plants in the field [59] and in those stored in closed containers at room temperature [59,63], in paper bags at room temperature [64,65], dry at ambient room temperature and relative humidity (RH) [66], at 30% RH at room temperature [67] and in sealed Petri dishes at 20 °C [68]. Also, afterripening is known to occur in seeds stored dry at low temperatures, for example, seeds of *Hordeum vulgare* L. afterripened at 8 °C [69], *Berberis* spp. at 3–4 °C [70], *Oryza sativa* L. (this is weedy rice, commonly called red rice, which is a variety of *O. sativa* that can be a serious pest in rice fields) at 5 °C [43], *Calendula* sp. at 4 °C [71], *Amaranthus retroflexus* L. at 0–5 °C [72], *Ochradenus baccatus* Delile at −18 °C [73], *Lepidium virginicum* L. at −20 °C [74], *Juniperus* sp. at −23 °C [75] and *Dactylis glomerata* L. at −75 °C [76]. However, seeds of *O. sativa* (weedy rice) did not afterripen when stored dry at −15 °C [43].

Afterripening in dry storage can be an important part of the dormancy-breaking protocol in seeds with the intermediate level of PD. For example, about 75–80% of the freshly matured seeds of *Isatis violascens* Bunge have intermediate PD. When seeds of this species were cold stratified at 4 °C immediately after collection, 21% of them germinated during a 12-week period at 4 °C. However, seeds allowed to afterripen in dry storage at room temperatures for six months germinated 100% during a 12-week period at 4 °C. Thus, 21% of the seeds had nondeep PD, and 79% had intermediate PD [77].

Afterripening is a part of the dormancy-breaking requirements of some seeds with morphophysiological dormancy (MPD). Seeds with MPD have a small underdeveloped embryo that grows inside the seed prior to radicle emergence (germination), and the embryo has PD. If the species is a winter annual and its seeds have nondeep simple MPD, PD is broken via afterripening during summer, and embryo growth and germination occur in autumn if exposed to light, as in *Chaerophyllum tainturieri* Hook. and Arn. [78] and *Papaver rhoeas* L. [79]. Afterripening is also a part of the dormancy-breaking requirements of some seeds with PY and a physiologically-dormant embryo, that is, seeds that have combinational dormancy (PY + PD). If the species is a winter annual and its seeds have PY + PD, PD is broken via afterripening during summer while the seed/fruit coat is water-impermeable. In autumn, the seed/fruit coat becomes water-permeable and the seed germinates, as in *Geranium carolinianum* L. [80] and *Vicia sativa* L. [81]. 

It should be mentioned that dry storage may, or may not, promote a dormancy-break in seeds with only PY. These seeds have a nondormant embryo but do not germinate due to a water-impermeable seed/fruit coat. During a long period of dry storage, some seeds with PY may become permeable, presumably due to opening of the water gap in the seed (or fruit) coat and not to afterripening per se. A water gap is a small structure in/on the seed (or fruit) coat of water-impermeable seeds (or fruits) that opens in response to appropriate environmental signals, thereby allowing water to enter [55]. After 12 years of dry storage at room temperatures, germination of *Mimosa foliolosa* Benth. seeds increased from about 10% to 60% but that of *M. maguirei* Barneby seeds decreased from about 10% to 5% [82]. After four years of dry storage at 15 °C and 15% RH germination of *Collaea argentina* Griseb. increased from 5% to 60% and that of *Abutilon pauciflorum* A.St.-Hil. seeds from 1% to 6% [83]. Seeds of wild *Glycine soja* Siebold and Zucc. were stored dry at room temperature for nine years, during which time water-permeable seeds increased from 4% to 91% in one ecotype and from 33% to 98% in a second ecotype [84]. Germination of *Astragalus cicer* L. seeds stored dry at room temperature for 14 years increased from 11% to 67% [85] but in another study, the germination (at 25 °C) of seeds of this species stored dry at room temperature for three years decreased from 18% to 11% [86].

## 3. Conditions Required for Afterripening

An early approach to studying afterripening was to store seeds at different RHs. Fresh (dormant) seeds of the winter annual *Draba verna* L. incubated over a range of RHs at 25 °C differed in the percentage of nondormant seeds after three months: 0–5% nondormant at 0–20% RH, 20–40% nondormant at 30–40% RH and 60–65% nondormant at 50–60% RH; seeds lost viability at 70–100% RH [87]. For the winter annual *Arabidopsis thaliana* (L.) Heynh., 33–75% RH (at 10 to 20 °C) was optimal for afterripening, while no afterripening occurred at 1% RH [88]. Most seeds of *Brassica japonica* Makino stored in a desiccator at room temperature were still dormant after three years, while 100% of the seeds stored in open air at room temperature were nondormant after two months [89].

Various studies of afterripening have now been done in which the seed moisture content (MC) was determined, and seeds were stored at specific temperatures and monitored until dormancy was broken. The idea behind these studies was to determine the optimum conditions for dormancy-break (Table 1). The most favorable conditions for afterripening often are a seed MC of 5%–10% fresh weight and temperatures of 20–30 °C, with storage time ranging from 1.5 to 12 months.

In general, afterripening during dry storage at low temperatures is slower than it is at high temperatures. Little afterripening occurred in seeds of *Oryza sativa* (weedy rice) at 5 °C, and it was faster at 30 °C than at 20 °C [43]. Similarly, only a few seeds of *Lolium rigidum* Gaudich. afterripened at 9 °C, and the order of afterripening at other temperatures was 50 > 40 > 30 > 20 °C [90]. The percentage of *Avena fatua* L. seeds that afterripened decreased with temperatures: 40 > 30 > 20 °C. However, as the temperature decreased the seed MC level required for afterripening to occur increased, for example, at 40 °C and 30 °C seed MC for afterripening was 10–12% and 14–20%, respectively [91]. However, for seeds of *Arabidopsis thaliana* with increased seed water content, the optimum temperature for afterripening increased, for example, at 0.04 and 0.08 g H_2_O [g dw]^−1^ the optimum temperature was 10 and 25 °C, respectively [88].

At a given temperature, afterripening usually is faster at relatively high than at low seed MC. At 30 and 45 °C, seeds of *Anthocercis littorea* Labill. with 5.0% MC afterripened faster than those with 2.9% MC, and seeds of *Dioscorea hastifolia* Nees with 9.7% MC afterripened faster than those with 5.3% MC [92]. At 23 °C, seeds of *Austrostipa elegantissima* (Labill.) S.W.L. Jacobs and Everett and *Conostylis candicans* Endl. equilibrated with 50% and 75% RH afterripened faster than those equilibrated with 5%, 13% or 23% RH [93]. 

As can be seen in Table 1, the rate (speed) at which seeds in dry storage become nondormant varies with species, MC and storage conditions. However, if we consider the life history of the species some general predictions can be made with regard to how long it may take seeds to become nondormant via afterripening. If species have seeds that normally undergo dormancy break when habitat temperatures are ≥ 15 °C, afterripening at room temperatures will be relatively rapid, that is, one to three months [59,63]. However, if species have seeds that normally undergo dormancy break when habitat temperatures are low enough for cold (moist) stratification (about 0 to 10 °C) afterripening is very slow. For example, seeds of the summer annual *Ambrosia trifida* L. mature in autumn, and cold stratification during winter breaks dormancy, with germination occurring in early spring. After three months of cold stratification at 5 °C, 100% of embryos removed from seeds of this species germinated, but only 5% of embryos removed from seeds dry-stored in the laboratory for six months did so [94].

## 4. What Happens to Seeds during Afterripening?

Various studies have been conducted in an attempt to understand why afterripened seeds germinate at higher percentages/rates than fresh seeds. For example, as the afterripening period for *Arachis hypogaea* L. seeds stored dry at room temperature increased ethylene production (at 48 h after the start of imbibition) and germination percentage increased [104]. On the other hand, seeds of *Nicotiana tabacum* stored dry at room temperature for one year exhibited faster testa and endosperm rupture than non-afterripened seeds [105].

Many studies have monitored changes in biochemistry and molecular biology of seeds during the afterripening period [106]. Bazin et al. [107] found that 24 of the mRNAs produced during seed development of *Helianthus annuus* L. and stored in the dry seeds were oxidized during afterripening at 5%, 60% and 75% RH at 25 °C. Oxidation of mRNAs resulted in changes in the transcription of genes involved in cell signaling and responses to stress. Further, the decrease in dormancy during dry storage of *H. annuus* seeds at 25 °C was correlated with a reduction in sensitivity to abscisic acid (ABA) [108]. In one genotype of *H. annuus*, ABA levels of afterripened seeds decreased when seeds were imbibed, and sensitivity to paclobutrazol (a plant growth retardant that inhibits gibberellin biosynthesis) and applied GA_3_ increased [108]. As seeds of *Triticum aestivum* L. afterripened, they first became sensitive to treatment with GA, which promoted germination, and then they became insensitive to ABA, an inhibitor of germination [109].

As seeds of *Helianthus annuus* afterripened at 5% and 75% RH at 25 °C for three and six weeks, reactive oxygen species (ROS) accumulated in cells of the embryonic axis, and during this time oxidation of specific embryo proteins and lipid peroxidation occurred [110]. ROS production also occurs during afterripening of seeds of other species, for example, *Hordeum vulgare* L. [111] and *Arabidopsis thaliana* [112]. Further, after nondormant seeds imbibe additional ROS are generated and play a role in controlling the germination process [113]. However, if seeds are stored for a long period of time they may lose viability due to the accumulation of ROS and a decrease in the antioxidant potential of cells in the embryo [114]. 

Many approaches are being taken to better understand the molecular biology of afterripening, including studies on gene transcription [115,116], genome expression of *Arabidopsis* mutants [117], mRNA expression profiling [118], proteomic analysis [119] and level of *DELAY OF GERMINATION 1* proteins [120]. For example, upon hydration of dormant and afterripened (nondormant) seeds of *Arabidopsis thaliana* differences were found in the gene expression profiles within three hours after sowing, indicating that “after-ripening presets the transcriptional response following initiation of imbibition” [116]. During afterripening of *A. thaliana* seeds with the GA-insensitive *sleepy 1-2* mutant that promotes dormancy, there was a reduction in dormancy-promoting transcripts of this gene stored in the seeds, which lead to breaking of dormancy [121]. Although much progress has been made in understanding afterripening, Chahtane et al. [122] concluded that still “primary seed dormancy and its regulation during after-ripening remain poorly understood”.

## 5. Potential Problems with Afterripening of Seeds

First, seeds may not afterripen in dry storage. Only a few (4–6%) seeds of *Corispermum lehmannianum* Bunge stored in paper bags at room conditions (20 °C, 20–30% RH) for seven months germinated when tested over a range of alternating temperature regimes [123]. Seeds of *Penstemon gibbensii* Dorn stored dry at room temperatures for zero and two years germinated to 16.3% and 15.3%, respectively, indicating that no afterripening occurred [124]. When seeds from six populations of *Sporobolus phleoides* Hack. ex Stucky. were stored dry at 20 °C for 0, 12, 18 and 24 months and then tested at 20, 30 and 30/20 °C, only seeds from two of the populations exhibited significant dormancy break. For the two populations with seed afterripening, however, maximum germination (after 12–18 months) was only 31% and 39% [125]. Further, in Asteraceae species with trimorphic diaspores, that is, three kinds of achenes in a capitulum that differ in size, mass and morphology, the amount of afterripening varies with the position of the achenes in the capitulum. Maximum germination of central, intermediate and peripheral achenes of *Garhadiolus papposus* Boiss and Buhse stored dry at room temperature for three months was 58%, 40% and 0%, respectively; peripheral achenes were viable [126].

Second, whereas seeds kept in conditions suitable for afterripening may become nondormant, and after an extended period they may exhibit symptoms of aging (decreased vigor) and eventually die (Table 2). Thus, seeds with a relatively high MC stored at high temperatures may afterripen quickly, but after six or more months of storage, depending on the species, they may be dead.

Third, seeds kept in conditions suitable for afterripening for an extended period may enter secondary dormancy. About 90% of seeds from each of two New Zealand populations (Oruawairua Island and Lincoln College) of *Arthropodium cirrhatum* (G. Forst.) R.Br. stored dry at room temperature for 6 months were nondormant; however, after 15 months of storage, about 50% and 98% of the seeds were nondormant, respectively. That is, a high proportion of the seeds from the Oruawairua Island population were viable but had entered secondary dormancy [127]. Achenes of *Cirsium arvense* (L.) Scop. with 6% MC stored at 12, 19, 26 and 33 °C for 0, 10, 30 and 150 days exhibited a decrease in germination from about 80% prior to storage to about 5% after storage at all four temperatures; seeds were viable [128]. After four months of storage in plastic containers at room temperature (20 to 25 °C) with 30% to 50% RH, seeds of *Amaranthus tuberculatus* (Moq.) Sauer collected in 2009 afterripened, with germination increasing from about 10% to 100%. After six months of storage, however, germination had decreased to 40%, indicating induction of secondary dormancy in the viable seeds [129]. Seeds of *Arabidopsis thaliana* stored for nine weeks at 33% and 45% RH at 10 °C; 45% and 56% RH at 15 °C, 56% RH at 20 °C; or 56%, 75% and 85% RH at 25 °C exhibited a significant decrease in germination when subsequently incubated at 25 °C, that is, secondary dormancy had been induced in the viable seeds [88]. 

Seeds of *Agriophyllum squarrosum* (L.) Moq. afterripened during dry storage at room temperature for one month [130,131], but in another study seeds of this species stored at room temperature for six months exhibited a decrease in germination from 92% to 39% [132], due to induction of secondary dormancy. In a third study, however, seeds stored at room temperature for 2–3 months and then stored in a refrigerator for four months germinated to 100% [133]. Thus, to ensure that seeds of this species will germinate when sown in spring the authors recommended: (1) store fresh seeds dry at room temperature for two to three months to allow afterripening to occur, and (2) transfer seeds to 4–5 °C to prevent them from entering secondary dormancy [134].

An extended period of dry storage at low temperatures can increase dormancy in seeds of some species. Dry storage of *Corylus avellana* L. nuts decreased the germination of embryos removed from the nuts. Whereas embryos from fresh nuts of this species germinated to 64% at 20 °C, those from nuts dry-stored at 10 °C for four and eight weeks germinated to only 30% and 10%, respectively [135]. Germinability of *Picea glauca* (Moench) Voss seeds with an MC of 5.5% was 87%, but after storage at 4, −20, −80 and −196 °C for six months, they germinated to only 52%, 47%, 36% and 23%, respectively. However, when seeds dry-stored for 6 months were cold stratified for 21 days germination was 89% to 91% [136]. Germination of *Alyssoides utriculata* (L.) Medik. and *Matthiola sinuata* (L.) R.Br. with an MC of 0.3% to 3% stored in sealed vials at –5 to –10 °C for 38–40 years decreased from 100% to 5% and 4%, respectively; 95% and 99% of these seeds were viable. However, seeds of 10 other species of Brassicaceae exposed to the same conditions germinated to 76–100% after the same period of storage, indicating no induction into secondary dormancy [137].

## 6. Management of Afterripening 

Since a high percentage of the species that might be used in restoration projects have nondeep PD, it is reasonable to think that careful manipulation of such seeds during storage would result in them becoming nondormant via afterripening. Therefore, nondormant seeds would be available for sowing. However, the phrase “careful manipulation” is important here. First seeds need to be stored under conditions that promote afterripening. Seed MC may need to be regulated prior to and during storage to avoid storing seeds with so little water in them that afterripening does not occur [88,89,90]. On the other hand, storing seeds at a high MC (or high RH) at a relatively high temperature might result in rapid afterripening, but it could also lead to seed death if storage under these conditions is prolonged (Table 2). If a restoration project involves numerous species, the suggested starting point for afterripening is to store seeds at room temperature at about 50% RH and a seed MC of 5–10%.

The recommendation is to determine seed MC prior to placing seeds into storage using the oven-drying method. If seeds have a MC well above or below the desired level, then adjustments in the MC need to be made. If the MC is above the desired 5%–10%, then seeds should be allowed to dry to this level. Large quantities of seeds can be dried in electric air-flow driers, preferably at a relatively low temperature. Seeds of *Carthamus tinctoris* were dried in an electric drier at 40, 50, 60 and 70 °C to 6.6% MC and then stored dry in sealed containers at room temperature for 240 days. Those dried at 40 °C had the highest viability [141]. 

If seeds have a MC below the desired level, the MC can be increased by allowing seeds to equilibrate in an atmosphere with a high RH (eRH). Studies of various species have been done in which seeds were allowed to equilibrate over a range of RHs, and then the MC of the seeds at each RH was determined. For small batches of seeds, a range of RHs can be obtained by using saturated salt solutions: lithium chloride (11% RH), magnesium chloride (32% RH), calcium nitrate (50% RH) and sodium chloride (75% RH) [142]. At a given RH, for example at 50% RH, the MC of seeds will vary, depending on the species (Table 3). 

It is important to conduct germination tests on freshly-matured seeds and at regular intervals test seeds stored under afterripening conditions [147]. Ideally, seeds should be tested at several alternating temperature regimes in both the light and dark, but it is especially important to test them at a temperature regime that simulates conditions in the field at the time the seeds will be sown. By monitoring seed germination at regular intervals, it will be apparent when dormancy has been broken. Thus, if seeds are still dormant (or cannot germinate at simulated field temperatures) at the time seed-sowing should take place in the field, then perhaps the seeds should not be used/sown. That is, if seeds are not capable of germinating in a Petri dish at simulated field temperatures (at the desired time of sowing), we should not expect them to germinate if they are sown in the field, where moisture stress is likely to occur. It would be better to delay sowing these seeds until they become nondormant than to risk having dormant seeds die or be eaten (or otherwise lost from the viable seed population) in the field. On the other hand, the decision might be made to allow dormancy-break to occur in the field under natural conditions. In the seasonally dry tropical forests of Northern Thailand, however, sowing seeds as soon as they matured vs. storing and then sowing them at the beginning of the rainy season did not significantly affect the number of seedlings established per 100 seeds sown [21]. It was concluded that species selection was more important than the time of seed sowing.

If dormancy is broken during dry storage several months prior to the time seeds will be sown in the field, then consideration should be given to transferring seeds to low temperatures (4–5 °C), hopefully, to prevent their entrance into secondary dormancy and/or loss of viability. We know that transferring nondormant seeds of *Agriophyllum squarrosum* to a low temperature prevents them from entering second dormancy prior to spring sowing [134] but it is not known how well this strategy would work for other species, for example, winter annuals. If fully afterripened seeds are transferred to a low temperature, continued germination monitoring is recommended to determine if seeds remain nondormant and viable. 

## 7. Future Research Needs 

One important question concerns the storage of seeds after they have become nondormant via afterripening. We have seen (above) that prolonged storage in conditions that promote afterripening can result in seeds entering secondary dormancy or even losing viability. After seeds have afterripened, would a decrease in MC to about 1% and/or a decrease in temperature prevent the seeds from entering secondary dormancy, or at least decrease the rate at which this happens? This is a question worthy of future research. 

If seeds afterripen and then subsequently are induced into secondary dormancy, what conditions are required to break secondary dormancy? In general, we do not have a good answer to this question, which could very likely vary with the species. In the case of *Arabidopsis thaliana* seeds stored at 10, 15, 20 and 25 °C at 45% RH, maximum germination of seeds tested at 25 °C occurred after seven weeks of storage. After nine weeks of storage, most of the seeds failed to germinate at 25 °C; they had been induced into secondary dormancy. However, continued storage at the four temperatures for up to 28 weeks resulted in an increase in the germination of seeds at 25 °C, but maximum germination was only about 35% [88].

Is there year-to-year variation in the ability of seeds of a particular species to afterripen? Seeds of *Amaranthus tuberculatus* collected in 2009 afterripened (100% germination) when stored dry at room temperatures for six months. However, seeds of this species collected in 2010 and stored for six months germinated to only 20% [129]. If there is year-to-year variation in the ability of seeds to afterripen in dry storage, how do environmental conditions under which the seeds matured differ between the years when seeds will and will not afterripen? Also, what role does the maternal environment play in the potential for seeds to be induced into secondary dormancy? Seeds of *Arabidopsis thaliana* produced at a low (14 °C) temperature were more likely to be induced into second dormancy by incubation under high water stress than those produced at a high (25 °C) temperature [148].

There is some evidence for intrataxon variation in how well seeds afterripen. After 70 days of dry storage at 35–40 °C, caryopses of *Hordeum spontaneum* K.Koch from six sites in Israel germinated to about 75%, 70%, 50%, 20%, 45% and 75% [149]. There is also a year-to-year variation in intrataxon variation in how well seeds afterripen. After 11 months of afterripening in dry storage at 23 °C, seeds of *Arabidopsis lyrata* (L.) O’Kane and Al-Shehbaz collected from three sites in 2007 germinated to 70%, 46% and 38%, respectively, but seeds collected in 2008 germinated to 60%, 13% and 25%, respectively [150].

## 8. Summary and Concluding Remarks

In summary, large quantities of seeds of native species are needed for restoration projects, but usually there are not enough seeds to meet the demand. Further, the expenses involved in propagating plants in greenhouses/nurseries and then transplanting them to the field suggest that it would be more cost-effective to sow seeds directly at the restoration sites. Sowing seeds in the field has been tried, but in many (but not all) cases percentages of germination/seedling establishment are very low. However, sowing nondormant seeds in the field during the natural germination season shows some promise for the establishment of a large number of seedlings at the site. A big challenge for people doing restoration is how to break dormancy in large quantities of seeds without having to use elaborate procedures. Fortunately, for people involved in the restoration of natural habitats seeds of many plant families desirable for re-introduction to field sites have nondeep physiological dormancy and will become nondormant during dry storage at room temperatures (afterripening). 

Thus, we have reviewed the temperature, seed moisture content and time required for afterripening to occur. Our aims were to make this information available to people who wish to break seed dormancy via afterripening and provide a better understanding of this method to break seed dormancy. This review also has emphasized that afterripening of seeds needs to be carefully monitored so that seeds can be removed from afterripening conditions after they become nondormant and before they die or enter secondary dormancy. Finally, we emphasize that much remains to be learned about seed afterripening, for example, the genes and metabolic pathways that account for dormancy break are not well known. Also, various ecological factors, such as the role of the maternal environment during seed development on afterripening requirements, have received little research attention. Clearly, much additional research needs to be done with regard to the afterripening of seeds.

## Figures and Tables

**Table 1:** The most effective conditions for afterripening of seeds of various species. Authorities are given only for species not mentioned in the text.

Species	Moisture Content (%)	Temperature (°C)	Time	References
*Austrostipa elegantissima*	7.1–13.2	23	6 mo	[93]
*Avena fatua*	10–20	40	3 mo	[91]
*Bromus tectorum*	7-8	20, 30	4 mo	[95]
*Carthamus tinctorius*	7.2–8.8	24.7	8 mo	[96]
*Conostylis candicans*	11.8	23	36 mo	[93]
*Euphorbia esula* L.	2.6	30	4–8 mo	[97]
*Helianthus annuus*	0.05 ^a^	20	1 wk	[98]
	0.04 ^a^	15, 20	3 wk	
*Heteropogon contortus* (L.) P.Beauv. ex Roem. and Schult.	5.7	30	12 mo	[99]
*Hordeum vulgare*	12	38	4 mo	[100]
	12	27	6 mo	
*Oryza sativa* (weedy rice)	6–14	25	1.5 mo	[101]
*Oryza sativa* (weedy rice)	10.8	30	2 mo	[102]
*Physaria fendleri* (A. Gray) O’Kane and Al-Shehbaz	4.75	35	3 mo	[103]
*Stylidium affine* Sond.	5.0–6.1	23	3 mo	[93]
	8.1–12.1	23	3 mo	
*Zygophyllum fruticulosum* DC.	5.7	30	12 mo	[92]
	5.7	45	10 mo	

^a^ g H_2_O [g dw]^−1^.

**Table 2:** Storage conditions that promoted afterripening, but a prolonged period at these conditions resulted in seed death. Authorities are given only for species not mentioned in the text.

Species	Moisture Conditions	Temperature (°C)	Time to Death (months)	References
*Arabidopsis thaliana*	75% RH	10, 15, 20, 25	15.8	[88]
*Draba verna*	70–100% RH	25	6	[87]
*Euphorbia esula*	≥ 9% MC	30	6	[97]
*Euphorbia heterophylla* L.	10.8% MC	25	6	[138]
	18.6% MC	25	3	
	18.6% MC	5	6	
*Heteropogon contortus*	75% eRH ^a^	20, 30	6	[99]
*Hordeum vulgare*	12% MC	38	5.3	[100]
	9.4% MC	38	8.3	
	10.3% MC	38	6–7.5	
*Leucocoryne* spp.	room conditions	room	72	[139]
*Salsola vermiculata* L.	9.6% MC	24	24	[140]

^a^ Seeds equilibrated with 75% RH.

**Table 3:** Some examples of final % seed MC after seeds were allowed to equilibrate with 40% and 50% RH. Authorities are given only for species not mentioned in the text.

	% Seed MC after Equilibration at		
Species	40% RH	50% RH	References
*Amaranthus tricolor* L.	8.95	10.28	[143]
*Apium graveolens* L.	6.79	7.82	[143]
*Beta vulgaris* L.	8.97	10.30	[143]
*Brassica juncea* (L.) Czern.	- ^a^	5.08–5.58	[142]
*Brassica napus* L.	- ^a^	4.91–5.84	[142]
*Brassica nigra* (L.) Koch	6.81	7.85	[143]
*Chenopodium album* L.	8.66	9.95	[143]
*Chenopodium foliosum* Asch.	9.30	10.67	[143]
*Dahlia pinnata* Cav.	7.29	9.10	[143]
*Daucus* carota L.	7.76	8.92	[143]
*Eruca sativa* Mill.	- ^a^	5.37–5.71	[142]
*Fraxinus pennsylvanica* Marshall	7.14	7.86	[144]
*Hordeum vulgare*	9.35	10.73	[143]
*Lolium rigidum*	8.89	10.0	[90]
*Oryza sativa*	10.0	11.88	[145]
*Pinus palustris* Mill.	6.75	7.63	[146]
*Zea mays* L.	9.04	10.37	[143]

^a^ no data.
